# Real-world clinical outcomes of cabozantinib as a second-line treatment for advanced hepatocellular carcinoma: a retrospective US claims analysis

**DOI:** 10.1093/oncolo/oyaf252

**Published:** 2025-08-09

**Authors:** Daniel H Ahn, Noh Jin Park, Michael Locker, Zheng-Yi Zhou, Xiaoyu Nie, Travis Wang, Shengsheng Yu

**Affiliations:** Department of Medical Oncology, Mayo Clinic, Phoenix, AZ 85054, United States; Department of Medical Affairs, Exelixis, Inc., Alameda, CA 94502, United States; Department of Medical Affairs, Exelixis, Inc., Alameda, CA 94502, United States; Analysis Group, Inc., Boston, MA 02199, United States; Analysis Group, Inc., Boston, MA 02199, United States; Analysis Group, Inc., Boston, MA 02199, United States; Department of Medical Affairs, Exelixis, Inc., Alameda, CA 94502, United States

**Keywords:** cabozantinib, hepatocellular carcinoma, retrospective studies, treatment outcome

## Abstract

**Background:**

Cabozantinib is indicated for hepatocellular carcinoma (HCC) following first-line (1L) sorafenib, but the 1L standard has shifted to immuno-oncology (IO)-based regimens. This study evaluated real-world outcomes among patients with advanced HCC receiving second-line (2L) cabozantinib following 1L therapies, including newer regimens.

**Patients and Methods:**

US claims data were used to identify adults with advanced HCC initiating 2L cabozantinib monotherapy (index date). Patients were stratified into three 1L treatment cohorts: IO monotherapy/IO + IO combination therapy; IO + non–IO combination therapy; or tyrosine kinase inhibitor (TKI) monotherapy. Real-world time to treatment discontinuation (rwTTD), real-world time to next treatment or death (rwTNTD), real-world overall survival (rwOS), and cabozantinib dosing were assessed collectively and by cohort. Adverse events were assessed before/after cabozantinib initiation.

**Results:**

Among 148 patients who received 2L cabozantinib, 28 were in the IO monotherapy/IO + IO combination therapy cohort, 54 in the IO + non–IO combination therapy cohort, and 66 in the TKI monotherapy cohort. Median rwTTD among all patients was 3.2 months; median rwTNTD was 7.6 months; 12-month rwOS rate was 61.6%. There were no significant differences in these outcomes among the three cohorts. Overall, 44.6% of patients initiated 2L cabozantinib at 60 mg/day, of whom 39.4% required a dose reduction; 37.8% initiated at 40 mg/day, of whom 16.1% had a dose reduction. Adverse event rates were similar before/after cabozantinib initiation.

**Conclusions:**

Cabozantinib shows consistent effectiveness and safety in the 2L HCC setting following prior TKIs or IO-based regimens in real-world clinical practice. These findings may inform 2L treatment decisions.

Implications for Practice In this retrospective study of patients with advanced hepatocellular carcinoma (HCC), real-world time to treatment discontinuation, real-world time to next treatment or death, and real-world overall survival for second-line (2L) cabozantinib were consistent regardless of first-line (1L) therapy with tyrosine kinase inhibitors or immuno-oncology–based regimens. As the 1L HCC treatment landscape continues to expand beyond the historically limited options, the current study findings can be used to inform 2L treatment decisions for patients with advanced HCC regardless of the 1L therapy.

## Introduction

Patients with advanced hepatocellular carcinoma (HCC) are typically treated with systemic therapies, with the tyrosine kinase inhibitor (TKI) sorafenib being the first to be approved in 2007 by the United States Food and Drug Administration for the first-line (1L) treatment of advanced HCC.^1^ A second TKI, lenvatinib, was only subsequently approved in 2018.^2^ More recently, the standard of care for 1L advanced HCC has shifted to immuno-oncology (IO)-based regimens, including atezolizumab (anti–programmed death-ligand 1) + bevacizumab (anti–vascular endothelial growth factor), approved in 2020, durvalumab (anti–programmed death-ligand 1) + tremelimumab (anti–cytotoxic T-lymphocyte associated protein 4), approved in 2022, and nivolumab (anti–programmed death-1) + ipilimumab (anti–cytotoxic T-lymphocyte associated protein 4), approved in 2025.^2–4^

Cabozantinib is an oral TKI that targets multiple receptor kinases, including vascular endothelial growth factor receptor, MET, and AXL.^5^ It was approved by the Food and Drug Administration in 2019 for the treatment of patients with HCC who have been previously treated with sorafenib, based on the CELESTIAL trial, where cabozantinib was associated with a significantly longer median overall survival (OS) (10.2 months vs 8.0 months, *P* = .005) and median progression-free survival (PFS) (5.2 months vs 1.9 months, *P* < .0001) compared with placebo.^5^^,^^6^

With the rapid expansion of the 1L HCC treatment landscape in the past few years, particularly IO-based regimens in the 1L setting, it is important to understand the real-world clinical and safety outcomes of second-line (2L) cabozantinib following 1L therapies other than sorafenib. Prior real-world studies of cabozantinib have primarily included patients previously treated with sorafenib, have not evaluated differences in cabozantinib outcomes by different types of 1L regimens, or have evaluated cabozantinib most frequently in the third line (3L) or later.^7–12^ Therefore, this study was conducted to understand real-world clinical and safety outcomes of 2L cabozantinib among patients with advanced HCC following various 1L therapies in the United States.

## Material and methods

### Data source

The study used insurance claims data from January 2017 to August 2023 from Komodo Health, Inc. (“Komodo data”; © 2025 Komodo Health, Inc. All rights reserved. Reprinted with permission). Komodo collects closed claims data directly from more than 150 payers in the United States, representing over 165 million lives, as well as data from multiple claims processors, to capture a total of 330 million unique patient journeys. For this study, the closed claims from the Komodo data were linked to patient mortality data via Datavant tokenization.

The data were deidentified and compliant with the Health Insurance Portability and Accountability Act of 1996; thus, no ethics board review was required. At the time of study conduct, all authors were either employed by or received research/consulting funding (work for hire) from Exelixis, Inc., the manufacturer of cabozantinib.

### Study design

A retrospective cohort study design was used (Figure S1). The index date was defined as the initiation date of 2L cabozantinib. The baseline period spanned from 6 months before the initiation of 1L systemic therapy to the index date (excluding the index date). The follow-up period was defined as the period from the index date to the earliest of the end of data availability, end of continuous insurance plan enrollment, or death.

**Figure 1.**
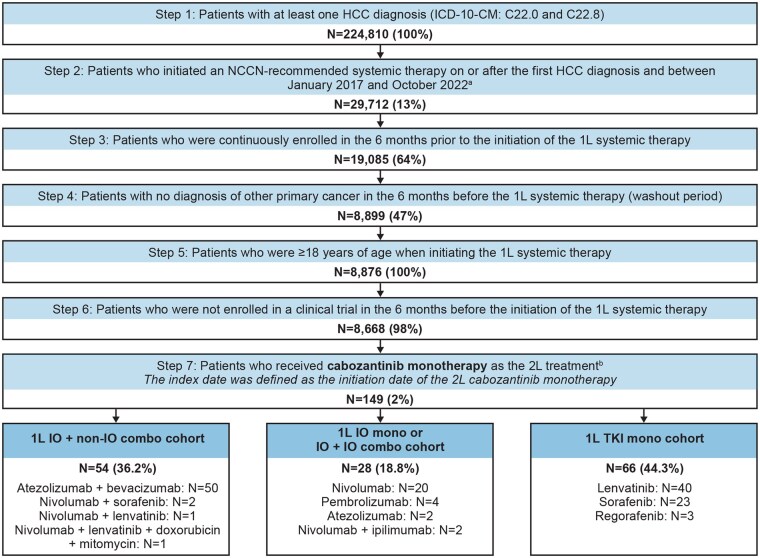
Sample selection. ^a^NCCN-recommended systemic therapies include atezolizumab + bevacizumab, sorafenib, lenvatinib, durvalumab, pembrolizumab, nivolumab, regorafenib, cabozantinib, ramucirumab, nivolumab + ipilimumab, dostarlimab-gxly, and selpercatinib.^13^ Some of these regimens are only recommended as subsequent-line systemic therapy options in the NCCN guidelines. However, whether a regimen was received in a line of therapy consistent with NCCN recommendations was not a selection criterion of our study sample. No patient in the data received tremelimumab, since the study period barely overlaps with the approval date of durvalumab + tremelimumab by the Food and Drug Administration (i.e., October 21, 2022). ^b^One patient who received ramucirumab monotherapy in 1L was excluded from the final sample. Abbreviations: 1/2L, first/second line; combo, combination therapy; HCC, hepatocellular carcinoma; ICD-10-CM, International Classification of Diseases, Tenth Revision, Clinical Modification; IO, immuno-oncology; mono, monotherapy; NCCN, National Comprehensive Cancer Network; TKI, tyrosine kinase inhibitor.

Lines of therapy (LOTs) were identified during the time from the initiation of the 1L systemic therapy to the earliest date of the end of continuous enrollment, end of data availability, or death. Treatment regimens (monotherapy or combination therapy) were defined as all drugs observed within 30 days of the first observed drug in a specific LOT. Switching to a new drug or add-on of a new drug after the 30-day window triggered a new LOT. Discontinuation of a LOT was defined by either a treatment gap of ≥60 consecutive days, death before or <60 days after the last day of supply of all drugs, or initiation of a new LOT. The discontinuation date was defined as the earliest of the last day of supply of all drugs in a LOT, the day before initiating the next LOT, or death.

### Patient selection

Patients were included in the study if they (1) had at least one HCC diagnosis (International Classification of Diseases, Tenth Revision, Clinical Modification [ICD-10-CM] codes: C22.0 and C22.8); (2) initiated at least one systemic therapy option recommended by the National Comprehensive Cancer Network Clinical Practice Guidelines in Oncology (NCCN Guidelines) for Hepatobiliary Cancers after the initial HCC diagnosis and between January 2017 and October 2022^13^; (3) were continuously enrolled during the 6 months prior to the initiation of the 1L systemic therapy; (4) had no diagnosis of other primary cancer (excluding nonmelanoma skin cancer, other specified carcinomas of liver, and unspecified liver carcinomas) in the 6 months prior to the initiation of the 1L systemic therapy (to ensure HCC-specific treatment); (5) were at least 18 years of age when initiating the 1L systemic therapy; (6) were not enrolled in a clinical trial during the 6 months before the initiation of the 1L systemic therapy (to ensure patients were not treated with investigational agents, as this study focused on the real-world management of HCC); and (7) received cabozantinib monotherapy as the 2L treatment (Figure 1).

Included patients were further divided into the following three cohorts based on their 1L treatments: (1) IO monotherapy or IO + IO combination therapy (“IO mono or IO + IO combo cohort”), (2) IO + non–IO combination therapy (“IO + non–IO combo cohort”), or (3) TKI monotherapy (“TKI mono cohort”).

### Study measures and statistical analysis

Patient baseline characteristics, including demographics, National Cancer Institute Comorbidity Index (NCICI), HCC-related comorbidities, and treatment history, were summarized and compared between the IO mono or IO + IO combo cohort and the TKI mono cohort and between the IO + non–IO combo cohort and the TKI mono cohort. Means and standard deviations were reported for continuous variables; frequencies and proportions were reported for categorical variables. Two-sample *t*-tests were used to compare continuous variables and chi-squared tests (or Fisher’s exact tests) were used to compare categorical variables.

Real-world time to treatment discontinuation (rwTTD), real-world time to next treatment or death (rwTNTD), and real-world overall survival (rwOS) in all patients and each of the three cohorts were analyzed as measures of real-world effectiveness of 2L cabozantinib. When using real-world data, rwTTD measures how long patients stay on the index treatment, while rwTNTD describes how soon patients switch from the index treatment to the next treatment (potentially as a proxy for real-world PFS [rwPFS]).^14^^,^^15^ In our current study, rwTTD was defined as the time from the index date to the discontinuation of 2L cabozantinib (see *Study Design* section for definitions). Real-world time to next treatment or death was defined as the time from the index date to the initiation of 3L therapy or death due to any cause, whichever occurred first. Real-world overall survival was defined as the time from the index date to death due to any cause. Real-world time to treatment discontinuation, rwTNTD, and rwOS were evaluated using the Kaplan–Meier approach and compared using log-rank tests. Median time to event, along with the 95% confidence interval (CI), or landmark rates (if median estimates were uncertain or imprecisely estimated, e.g., due to a small number of patients at risk) were reported. Patients without events were censored on the date of the last follow-up/data availability or end of continuous eligibility, whichever occurred first.

The starting dose of 2L cabozantinib and proportions of patients with dose reduction from each starting dose were reported for all patients and each of the three cohorts. Dose was defined based on the strengths indicated by the National Drug Codes.^16^

The prevalence of adverse events (AEs), identified using ICD-10-CM codes, was assessed among all patients during the 6 months before the 1L systemic therapy, the 1L treatment period, and the 2L treatment period. All grade 3/4 AEs reported by at least one patient in the cabozantinib registrational Phase 3 CELESTIAL trial in HCC were measured in this study.^6^ Prevalence of AEs during each period was calculated as the number of patients with each AE divided by the total number of patients.

All analyses were conducted using SAS Enterprise Guide, version 8.3 (SAS Institute Inc.).

## Results

The study included 148 patients who received 2L cabozantinib monotherapy, of which 28 were included in the IO mono or IO + IO combo cohort, 54 in the IO + non–IO combo cohort, and 66 in the TKI mono cohort. The most common 1L regimen was nivolumab (71.4%) in the IO mono or IO + IO combo cohort, atezolizumab + bevacizumab (92.6%) in the IO + non–IO combo cohort, and lenvatinib (60.6%) in the TKI mono cohort (Figure 1).

### Baseline characteristics

The mean age of the overall population of patients who received 2L cabozantinib was 64.6 years (range across cohorts: 63.6-66.3 years), and 78.4% were male (range: 74.2%-83.3%; Table 1). The overall proportions of patients who initiated 2L cabozantinib increased over time, and the TKI mono cohort had a larger proportion of patients whose index dates were in 2020 or prior (60.6%) compared with the other two cohorts (53.6% in the IO mono or IO + IO combo cohort and 16.7% in the IO + non–IO combo cohort). Overall, the largest proportion of patients had commercial insurance coverage (29.1%), and patients were relatively evenly distributed across US geographic regions. The mean duration of follow-up after cabozantinib initiation was 12.4 months in the overall population, 16.7 months in the IO mono or IO + IO combo cohort, 10.4 months in the IO + non–IO combo cohort, and 12.2 months in the TKI mono cohort.

**Table 1.** Baseline demographic and disease characteristics.

	Overall population	**IO mono or IO** **+** **IO combo**	**IO** **+** non–**IO combo**	TKI mono
	**(*N*** **=** **148)**	**(*N*** **=** **28)**	**(*N*** **=** **54)**	**(*N*** **=** **66)**
**Age at HCC diagnosis (years), mean ± SD [median]**	64.6 ± 9.1 [63.9]	66.3 ± 11.5 [68.1]	65.0 ± 8.8 [63.8]	63.6 ± 8.1 [63.8]
**Male**	116 (78.4)	22 (78.6)	45 (83.3)	49 (74.2)
**Year of the index date**				
**2018**	7 (4.7)	1 (3.6)	0^a^	6 (9.1)
**2019**	26 (17.6)	9 (32.1)	2 (3.7)^a^	15 (22.7)
**2020**	31 (20.9)	5 (17.9)	7 (13.0)^a^	19 (28.8)
**2021**	40 (27.0)	7 (25.0)	22 (40.7)^a^	11 (16.7)
**2022**	44 (29.7)	6 (21.4)	23 (42.6)^a^	15 (22.7)
**Insurance type**				
**Commercial**	43 (29.1)	5 (17.9)	16 (29.6)	22 (33.3)
**Medicaid**	28 (18.9)	5 (17.9)	10 (18.5)	13 (19.7)
**Medicare**	33 (22.3)	6 (21.4)	12 (22.2)	15 (22.7)
**Multiple**	32 (21.6)	10 (35.7)	12 (22.2)	10 (15.2)
**Other**	8 (5.4)	2 (7.1)	3 (5.6)	3 (4.5)
**Unknown/missing**	4 (2.7)	0 (0.0)	1 (1.9)	3 (4.5)
**Region**				
**Midwest**	22 (14.9)	2 (7.1)	6 (11.1)	14 (21.2)
**Northeast**	24 (16.2)	8 (28.6)	5 (9.3)	11 (16.7)
**Southeast**	30 (20.3)	7 (25.0)	11 (20.4)	12 (18.2)
**Southwest**	26 (17.6)	3 (10.7)	14 (25.9)	9 (13.6)
**West**	18 (12.2)	2 (7.1)	8 (14.8)	8 (12.1)
**Multiple**	14 (9.5)	3 (10.7)	6 (11.1)	5 (7.6)
**Unknown/missing**	14 (9.5)	3 (10.7)	4 (7.4)	7 (10.6)
**Follow-up time (months), mean ± SD [median]**	12.4 ± 11.5 [8.7]	16.7 ± 13.4 [13.4]	10.4 ± 9.8 [8.0]	12.2 ± 11.7 [8.8]
**Duration of 1L (months), mean ± SD [median]**	5.7 ± 4.4 [4.1]	5.9 ± 4.9 [4.1]	5.8 ± 4.3 [4.2]	5.4 ± 4.3 [3.6]
**Metastatic disease during the baseline period**	72 (48.6)	15 (53.6)	27 (50.0)	30 (45.5)
**NCICI, mean ± SD [median]**	4.3 ± 2.4 [3.7]	4.6 ± 2.5 [4.8]	4.2 ± 2.4 [3.8]	4.1 ± 2.3 [3.4]
**HCC-related comorbidities during the baseline period**				
**HBV only**	11 (7.4)	2 (7.1)	2 (3.7)	7 (10.6)
**HCV only**	77 (52.0)	13 (46.4)	28 (51.9)	36 (54.5)
**Both HBV and HCV**	9 (6.1)	1 (3.6)	3 (5.6)	5 (7.6)
**Liver cirrhosis**	113 (76.4)	22 (78.6)	43 (79.6)	48 (72.7)
**Hypertension**	110 (74.3)	21 (75.0)	44 (81.5)	45 (68.2)
**Decompensated cirrhosis**	83 (56.1)	17 (60.7)	33 (61.1)	33 (50.0)
** Alcohol and drug use disorders**	69 (46.6)	12 (42.9)	32 (59.3)^a^	25 (37.9)
**Portal hypertension**	52 (35.1)	10 (35.7)	26 (48.1)^a^	16 (24.2)
**Esophageal varices**	43 (29.1)	5 (17.9)	20 (37.0)	18 (27.3)
**Other ascites**	44 (29.7)	12 (42.9)	16 (29.6)	16 (24.2)
**Thrombocytopenia**	38 (25.7)	8 (28.6)	15 (27.8)	15 (22.7)
**Splenomegaly**	39 (26.4)	6 (21.4)	18 (33.3)	15 (22.7)
**Portal vein invasion**	32 (21.6)	7 (25.0)	12 (22.2)	13 (19.7)
**NASH or NAFLD**	25 (16.9)	6 (21.4)	10 (18.5)	9 (13.6)
** Alcoholic cirrhosis/alcoholic hepatitis**	24 (16.2)	2 (7.1)	11 (20.4)	11 (16.7)
**Gastric varices**	9 (6.1)	1 (3.6)	6 (11.1)	2 (3.0)
**Treatment history during the baseline period**				
**TACE/TARE**	48 (32.4)	9 (32.1)	23 (42.6)	16 (24.2)
**Surgery**	4 (2.7)	2 (7.1)	0	2 (3.0)
**Liver transplant**	1 (0.7)	0	0	1 (1.5)

Data are *n* (%), unless otherwise specified.

a Indicates *P*-value <.05 compared with the TKI mono cohort.

Abbreviations: 1L, first line; combo, combination therapy; HBV, hepatitis B virus; HCC, hepatocellular carcinoma; HCV, hepatitis C virus; IO, immuno-­oncology; mono, monotherapy; NAFLD, non-alcoholic fatty liver disease; NASH, non-alcoholic steatohepatitis; NCICI, National Cancer Institute Comorbidity Index; SD, standard deviation; TACE, transarterial chemoembolization; TARE, transarterial radioembolization; TKI, tyrosine kinase inhibitor.

The mean duration of 1L treatment was 5.7 months overall (range: 5.4-5.9 months) and the mean NCICI score was 4.3 (range: 4.1-4.6). Approximately half of all patients (52.0%) had a hepatitis C virus (HCV) infection (range: 46.4%-54.5%). Fewer patients in the TKI mono cohort than the IO + non–IO combo cohort had alcohol and drug use disorders (37.9% vs. 59.3%, respectively) and portal hypertension (24.2% vs. 48.1%) during the baseline period (both *P* < .05; Table 1). No statistically significant differences were found between cohorts in other HCC-related comorbidities or treatment history during the baseline period.

### Clinical outcomes

**Figure 2.**
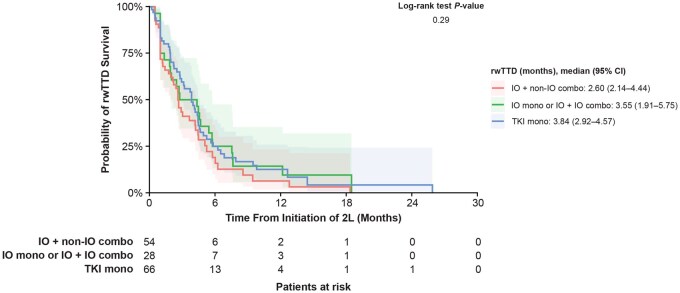
rwTTD of 2L cabozantinib stratified by 1L therapy. Abbreviations: 1/2L, first/second line; CI, confidence interval; combo, combination therapy; IO, immuno-oncology; mono, monotherapy; rwTTD, real-world time to treatment discontinuation; TKI, tyrosine kinase inhibitor.

The median rwTTD among all patients receiving 2L cabozantinib was 3.2 (95% CI: 2.7-4.3) months. There was no statistically significant difference in median rwTTD between the IO mono or IO + IO combo cohort (3.6 months; 95% CI: 1.9-5.8), the IO + non–IO combo cohort (2.6 months; 95% CI: 2.1-4.4), and the TKI mono cohort (3.8 months; 95% CI: 2.9-4.6; *P* = .29; Figure 2).

**Figure 3.**
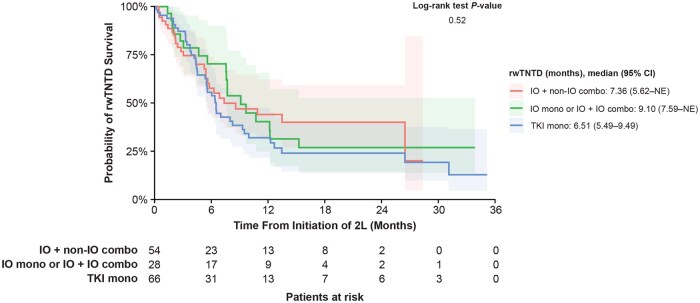
rwTNTD of 2L cabozantinib stratified by 1L therapy. Abbreviations: 1/2L, first/second line; CI, confidence interval; combo, combination therapy; IO, immuno-oncology; mono, monotherapy; rwTNTD, real-world time to next treatment or death; TKI, tyrosine kinase inhibitor.

The median rwTNTD among all patients was 7.6 (95% CI: 6.3-10.0) months. There was no statistically significant difference in median rwTNTD between the IO mono or IO + IO combo cohort (9.1 months; 95% CI: 7.6-not estimated [NE]), the IO + non–IO combo cohort (7.4 months; 95% CI: 5.6-NE), and the TKI mono cohort (6.5 months; 95% CI: 5.5-9.5; *P* = .52; Figure 3). The 12-month rwTNTD rate was 37.5% among all patients, 40.3% in the IO mono or IO + IO combo cohort, 44.0% in the IO + non–IO combo cohort, and 32.0% in the TKI mono cohort.

**Figure 4.**
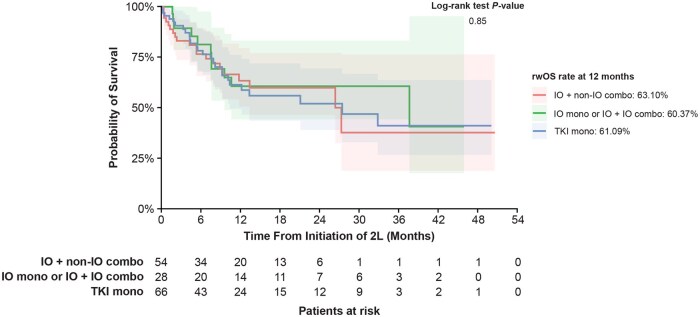
rwOS of 2L cabozantinib stratified by 1L therapy. Abbreviations: 1/2L, first/second line; combo, combination therapy; IO, immuno-oncology; mono, monotherapy; rwOS, real-world overall survival; TKI, tyrosine kinase inhibitor.

For rwOS, the 12-month rwOS rate was 61.6% among all patients, 60.4% in the IO mono or IO + IO combo cohort, 63.1% in the IO + non–IO combo cohort, and 61.1% in the TKI mono cohort (Figure 4). The differences between the cohorts were not statistically significant (*P* = .85).

### Dosing of 2L cabozantinib and prevalence of AEs

In the overall population, 66 (44.6%) patients initiated 2L cabozantinib at the 60-mg dose (range across cohorts: 40.9%-48.1%), 56 (37.8%) at the 40-mg dose (range: 33.3%-46.4%), and 26 (17.6%) at the 20-mg dose (range: 7.1%-21.2%; Table 2). The proportion of patients who experienced a dose reduction from a starting dose of 60 mg was 39.4%, ranging from 26.9% in the IO + non–IO combo cohort to 53.8% in the IO mono or IO + IO combo cohort. The proportion of patients who experienced a dose reduction from a starting dose of 40 mg was 16.1%, ranging from 7.7% in the IO mono or IO + IO combo cohort to 22.2% in the IO + non–IO combo cohort.

**Table 2.** Starting dose of 2L cabozantinib and dose reduction by cohort.

	Overall population	**IO mono or IO** **+** **IO combo**	**IO** **+** **non–IO combo**	TKI mono
	**(*N*** **=** **148)**	**(*N*** **=** **28)**	**(*N*** **=** **54)**	**(*N*** **=** **66)**
**Starting dose of 20 mg**	26 (17.6)	2 (7.1)	10 (18.5)	14 (21.2)
**Starting dose of 40 mg**	56 (37.8)	13 (46.4)	18 (33.3)	25 (37.9)
**Dose reduction from 40 mg**	9 (16.1)	1 (7.7)	4 (22.2)	4 (16.0)
**No dose reduction from 40 mg**	47 (83.9)	12 (92.3)	14 (77.8)	21 (84.0)
**Starting dose of 60 mg**	66 (44.6)	13 (46.4)	26 (48.1)	27 (40.9)
**Dose reduction from 60 mg**	26 (39.4)	7 (53.8)	7 (26.9)	12 (44.4)
**No dose reduction from 60 mg**	40 (60.6)	6 (46.2)	19 (73.1)	15 (55.6)

Data are *n* (%).

Abbreviations: 2L, second-line; combo, combination therapy; IO, immuno-oncology; mono, monotherapy; TKI, tyrosine kinase inhibitor.

Overall, AE rates were generally similar during the 6 months before 1L and the 1L and 2L treatment periods, with hypertension, abdominal pain, and anemia being the most commonly observed across various treatments received (Table 3). No new safety signals for cabozantinib were identified.

**Table 3.** AE prevalence during the 6 months before 1L therapy, the 1L treatment period, and the 2L treatment period.

	**During 6** **months before 1L**	During 1L	During 2L
	**(*N*** **=** **148)**	**(*N*** **=** **148)**	**(*N*** **=** **148)**
**Hypertension**	91 (61.5)	96 (64.9)	82 (55.4)
**Abdominal pain**	64 (43.2)	58 (39.2)	52 (35.1)
**Anemia**	50 (33.8)	45 (30.4)	46 (31.1)
**Fatigue**	25 (16.9)	38 (25.7)	42 (28.4)
**Ascites**	20 (13.5)	33 (22.3)	37 (25.0)
**Diarrhea**	9 (6.1)	27 (18.2)	36 (24.3)
**Thrombocytopenia**	21 (14.2)	27 (18.2)	34 (23.0)
**Nausea/vomiting**	18 (12.2)	35 (23.6)	33 (22.3)
**Dyspnea**	25 (16.9)	25 (16.9)	32 (21.6)
**Peripheral edema**	12 (8.1)	22 (14.9)	25 (16.9)
**Asthenia**	12 (8.1)	8 (5.4)	24 (16.2)
**Constipation**	22 (14.9)	29 (19.6)	22 (14.9)
**Bleeding/hemorrhage**	10 (6.8)	18 (12.2)	21 (14.2)
**Cough**	16 (10.8)	15 (10.1)	19 (12.8)
**Upper abdominal pain**	37 (25.0)	27 (18.2)	19 (12.8)
**Decreased appetite**	11 (7.4)	21 (14.2)	18 (12.2)
**Back pain**	21 (14.2)	18 (12.2)	13 (8.8)
**Mucosal inflammation**	12 (8.1)	9 (6.1)	13 (8.8)
**Rash**	4 (2.7)	13 (8.8)	11 (7.4)
**Dizziness**	7 (4.7)	10 (6.8)	8 (5.4)
**Increase in serum bilirubin level**	5 (3.4)	3 (2.0)	7 (4.7)
**Increase in aspartate/alanine aminotransferase level**	5 (3.4)	8 (5.4)	6 (4.1)
**Palmar-plantar erythrodysesthesia**	1 (0.7)	6 (4.1)	6 (4.1)
**Headache**	10 (6.8)	12 (8.1)	5 (3.4)
**Hypoalbuminemia**	1 (0.7)	2 (1.4)	5 (3.4)
**Stomatitis**	0	3 (2.0)	5 (3.4)
**Dysphonia**	0	2 (1.4)	1 (0.7)
**Insomnia**	0	0	1 (0.7)
**Weight loss**	3 (2.0)	0	0

Data are *n* (%).

Abbreviations: 1/2L, first/second line; AE, adverse event.

## Discussion

In this retrospective real-world study of patients with advanced HCC, clinical outcomes were examined in patients who received cabozantinib in the 2L setting. As expected in a real-world patient population, patients received a wide range of 1L regimens, including atezolizumab + bevacizumab, lenvatinib, and nivolumab before the initiation of 2L cabozantinib, and IO-based regimens were increasingly used in 1L over time. Clinical outcomes for 2L cabozantinib were consistent regardless of 1L therapy, with a median rwTTD of 3.2 months and rwTNTD of 7.6 months overall. The rate of rwOS was 61.6% at 12 months post-initiation of cabozantinib. In addition to clinical outcomes, safety outcomes, including dose reduction as an indicator of potential safety concerns, were evaluated. Dose reduction was required in less than 40% of patients at the highest starting dose of 60 mg, with 60.6% continuing on the 60-mg dose, which may reflect the variability of cabozantinib clearance between patients.^17^ Lastly, no new safety signals were identified. These findings confirm the effectiveness and safety of cabozantinib in 2L HCC following prior TKI or IO-based regimens.

Cabozantinib was approved for the treatment of HCC based on the randomized, placebo-controlled, Phase 3 CELESTIAL clinical trial.^5^^,^^6^ Since sorafenib was the standard of care for 1L HCC at the time of trial conduct, all included patients had received prior treatment with sorafenib, though 27% were also treated with an additional systemic agent for HCC (i.e., cabozantinib was received in the 3L setting).^6^^,^^18^ Despite the inclusion of patients who received previous treatment other than sorafenib in the current study, the findings were generally consistent with those of CELESTIAL. The median duration of receipt of cabozantinib in the clinical trial was 3.8 months, which was slightly longer than the rwTTD of 3.2 months in the present study. One potential explanation for this observation could be that the patients in CELESTIAL were allowed to continue cabozantinib beyond progression, per study protocol.^6^ As a proxy for rwPFS,^14^^,^^15^ the median rwTNTD of 7.6 months was longer than the median PFS of 5.2 months observed in the CELESTIAL trial. Similarly, among the patients who received 1L TKI monotherapy in the current study, the median rwTNTD (6.5 months) was slightly longer than the median PFS (5.5 months) reported among the subgroup of 331 patients in the cabozantinib arm of CELESTIAL who received sorafenib as the only prior systemic treatment.^18^ Notably, rwTNTD estimates may be larger than corresponding PFS estimates in settings with few therapeutic options and thus potential delays in initiation of subsequent therapy.^14^^,^^15^ Besides the usual differences between real-world practice and clinical trial settings, the discrepancies in outcomes may also be due to different patient characteristics between the two populations. For instance, the current patient sample had higher proportions of HCV only (52.0% vs. 24%) and dual hepatitis B virus (HBV)/HCV infection (6.1% vs. 2%) relative to that of the CELESTIAL trial, but lower HBV only infection (7.4% vs. 38%).^6^ Moreover, the present study focused on patients in the United States, whereas CELESTIAL included a global population. Lastly, the safety profile of 2L cabozantinib observed in the current study was generally consistent with that seen in the CELESTIAL trial.^6^

A more recent open-label, Phase 2 trial by Chan et al. of patients with HCC receiving cabozantinib following IO-based regimens found that among the subgroup of 27 patients treated with 2L cabozantinib, median PFS was 4.3 months,^19^ which is shorter than the rwTNTD of 7.4 months in the IO + non–IO combo cohort and 9.1 months in the IO mono or IO + IO combo cohort in the current study. Again, the discrepancy may be explained by differences in study settings, outcome definitions, patient characteristics (e.g., most patients in the clinical trial had HBV), and country of patient recruitment.

While prior real-world studies have described the clinical outcomes of cabozantinib among patients with HCC,^7^^,^^8^^,^^10–12^ there is limited evidence evaluating its use specifically in the 2L following different 1L treatments. A few studies have assessed clinical outcomes among patients who received cabozantinib after progression on prior HCC treatments, but the majority of included patients were treated with cabozantinib in the 3L or later.^7^^,^^8^^,^^12^ In these studies, median rwPFS from cabozantinib initiation ranged from 3.4 to 5.1 months.^7^^,^^8^^,^^12^ Two US-based studies evaluated patients who received 2L or later cabozantinib (majority 3L or later) after progression on IO and reported median rwPFS of 2.1 months.^10^^,^^11^ These rwPFS estimates are shorter than the median rwTNTD of 7.6 months reported in the current study, though the discrepancy may be due to the inclusion of patients receiving cabozantinib in later lines in prior studies. Moreover, the sample sizes of many of the aforementioned studies were quite small (*N* < 30), limiting their power and validity.^8^^,^^10^^,^^11^

With regard to rwTTD, a few prior analyses have evaluated similar or comparable outcomes in real-world clinical practice. Kanzaki et al. estimated time to treatment failure (as indicated by discontinuation) based on Kaplan–Meier analysis and reported medians of 2.1 months for 3L cabozantinib and 1.1 months for 4L cabozantinib.^8^ In a separate study of patients with HCC who received 3L or later cabozantinib after prior treatment with atezolizumab + bevacizumab, the median duration of cabozantinib based on Kaplan–Meier analysis was 1.6 months.^9^ Again, these estimates may not be directly comparable to the rwTTD of 3.2 months reported in the current study, since cabozantinib was used in different LOTs.

In summary, the current study findings suggest that clinical outcomes with cabozantinib treatment in 2L have remained relatively consistent since the drug’s approval for HCC in 2019, even with the significantly expanded 1L treatment landscape over the last few years. Indeed, no significant differences in clinical outcomes and treatment-associated toxicities were observed among patients receiving different 1L treatments prior to cabozantinib. These findings help to address the data gap for 2L treatment options after a non–sorafenib 1L therapy in real-world clinical practice. This point is further corroborated by a retrospective study of patients with advanced HCC receiving 2L or later cabozantinib by Bang et al., where there was no difference in rwPFS between patients with or without prior treatment of IO-based regimens (*P* = .43).^20^ Similarly, a subgroup analysis of the CELESTIAL trial demonstrated that patients treated with a prior IO agent and the required sorafenib had similar outcomes on cabozantinib relative to the overall group of patients with a variety of two prior regimens.^21^ Lastly, in the aforementioned Phase 2 trial by Chan et al., univariate and multivariable analyses indicated that line of therapy, and not type of prior therapy, was the only independent prognostic factor affecting OS.^19^ As the therapeutic landscape for HCC continues to grow, the present study findings may be used to inform treatment decisions in the 2L for patients with HCC in real-world clinical practice.

### Limitations

The results should be considered in the context of some limitations. Claims data are naturally subject to inaccuracies in coding and billing. Additionally, an observed prescription fill does not guarantee the medication was taken as prescribed. However, these limitations were expected to affect all cohorts relatively consistently. LOTs were defined using an algorithm, which may not have matched patients’ actual treatment history. Furthermore, patients’ clinical characteristics such as tumor stage and alpha-fetoprotein levels, severity of AEs, reasons for treatment discontinuation, and physicians’ rationales for dosage decisions are not available and thus, could not be described for each cohort. Moreover, potential underreporting of the mortality data may have impacted mortality-related outcomes. The sample sizes of the study cohorts were relatively small, which may have limited the ability to detect true clinical differences. Lastly, the results may not be generalizable to patients with traditional Medicare, the uninsured, or other patient populations with insurance for which the Komodo data do not have sufficient representation. Besides expanding the study to a broader patient population, future research may also consider stratifying the analyses of clinical outcomes and dosing by patient characteristics.

## Conclusions

Despite the claims-based and study limitations discussed above, this retrospective analysis demonstrated the consistent effectiveness and safety of cabozantinib in the 2L HCC setting following prior treatment with TKIs or IO-based regimens in the real world. rwTTD, rwTNTD (as a proxy of rwPFS), and rwOS following 2L cabozantinib treatment were consistent across different 1L therapies. As the 1L HCC treatment landscape continues to expand beyond the historically limited options, the current study findings can be used to inform 2L treatment decisions for patients with advanced HCC regardless of the 1L therapy.

## Supplementary Material

oyaf252_Supplementary_Data

## Data Availability

The data that support the findings of this study are available from Komodo Health. Restrictions apply to the availability of these data, which were used under license for this study. Data are available at https://www.komodohealth.com/ with the permission of Komodo Health.
