# Supplementary material for: Real-world clinical outcomes of cabozantinib as a second-line treatment for advanced hepatocellular carcinoma: a retrospective US claims analysis
Source: Oncologist. 2025 Aug 9;30(9):oyaf252. doi: 10.1093/oncolo/oyaf252 (PMC12445639; doi:10.1093/oncolo/oyaf252)
Supplement: oyaf252_Supplementary_Data [file oyaf252_supplementary_data.docx]

**Supplemental Figure 1. Study design**

End of data availability, continuous eligibility, or death

HCC diagnosis

1L systemic therapy

2L cabozantinib

**Index date**

6 months of continuous enrollment and no diagnosis of other primary cancer

**Follow-up period**

**Baseline period**

**Abbreviations**: 1/2L, first/second line; HCC, hepatocellular carcinoma.
